# Making sense of the French public hospital system: a network-based approach to hospital clustering using unsupervised learning methods

**DOI:** 10.1186/s12913-021-07215-4

**Published:** 2021-11-17

**Authors:** Jan Chrusciel, Adrien Le Guillou, Eric Daoud, David Laplanche, Sandra Steunou, Marie-Caroline Clément, Stéphane Sanchez

**Affiliations:** 1 Pôle Territorial Santé Publique et Performance, Centre Hospitalier de Troyes, F-10000 Troyes, France; 2 Pôle Recherche et Santé Publique, Centre Hospitalier Universitaire de Reims, 51100 Reims, France; 3 Residual Tumor & Response to Treatment Laboratory, RT2Lab, INSERM, U932 Immunity and Cancer, Institut Curie, Université Paris, 75005 Paris, France; 4 Department of Data, Agence Technique d’Information sur l’Hospitalisation, 69003 Lyon, France; 5 Department of Classifications in Healthcare, Medical Information and Financing Models, Agence Technique d’Information sur l’Hospitalisation, 75012 Paris, France

## Abstract

**Background:**

Hospitals in the public and private sectors tend to join larger organizations to form hospital groups. This increasingly frequent mode of functioning raises the question of how countries should organize their health system, according to the interactions already present between their hospitals. The objective of this study was to identify distinctive profiles of French hospitals according to their characteristics and their role in the French hospital network.

**Methods:**

Data were extracted from the national hospital database for year 2016. The database was restricted to public hospitals that practiced medicine, surgery or obstetrics. Hospitals profiles were determined using the k-means method. The variables entered in the clustering algorithm were: the number of stays, the effective diversity of hospital activity, and a network-based mobility indicator (proportion of stays followed by another stay in a different hospital of the same Regional Hospital Group within 90 days).

**Results:**

Three hospital groups were identified by the clustering algorithm. The first group was constituted of 34 large hospitals (median 82,100 annual stays, interquartile range 69,004 – 117,774) with a very diverse activity. The second group contained medium-sized hospitals (with a median of 258 beds, interquartile range 164 - 377). The third group featured less diversity regarding the type of stay (with a mean of 8 effective activity domains, standard deviation 2.73), a smaller size and a higher proportion of patients that subsequently visited other hospitals (11%). The most frequent type of patient mobility occurred from the hospitals in group 2 to the hospitals in group 1 (29%). The reverse direction was less frequent (19%).

**Conclusions:**

The French hospital network is organized around three categories of public hospitals, with an unbalanced and disassortative patient flow. This type of organization has implications for hospital planning and infectious diseases control.

**Supplementary Information:**

The online version contains supplementary material available at 10.1186/s12913-021-07215-4.

## Background

Coordination of hospital activity across multiple sites has been increasingly frequent in the United States and European countries since 1970 [1]. In various industries, the coordination of processes over multiple sites is seen as a way to improve performance. However, the extent to which this principle applies to healthcare has been debated [2, 3]. Integrated healthcare is increasingly seen as an attempt to improve healthcare quality [4]. Hospitals can assemble in informal networks or health systems having a common leadership [3]. These networks should offer accessible care, in a coordinated effort with a common information and quality insurance system with financial incentives [5]. Horizontal integration refers to organizations that acquire or integrate with other organizations that provide similar services, whereas vertical integration happens when the parties in question offer different level of care, services or functions [6]. Drawing on the example of the loss of market share of the Kaiser Permanenente HMO (Health Maintenance Organization), some authors have made the case for flexible organizations, with partners acting as equal team members rather than leaders and followers [2]. However, a strong hierarchical structure with clear targets has been shown to be efficient in helping regions to recover from poor hospital performance [7]. Overall, vertical integration of health systems is on the increase [8]. For example, the reduction in use of inpatient care and financial incentives in the Affordable Care Act have transformed the United States healthcare system from a variety of independent hospitals to a few locally integrated health care systems. The treatment of severe disease requires specialized infrastructure that is only found in large university hospitals, sometimes called tertiary care hospitals. Regionalization allows patients to get adequate care in these high-volume centers [9, 10], although the relationship between volume and quality has been debated [11]. These hospitals naturally assume the role of leaders in hospital systems. Following the trend towards increasing hospital system integration, nationwide reforms have taken place in several countries to restructure and coordinate hospital systems. In Denmark in 2007, a nationwide healthcare regionalization reform decreased the number of acute hospitals from 40 to 21, with encouraging results for productivity and quality of care, although the number of medical professionals also increased during this period [12]. Hospital networks have been introduced to Belgium in 2020, with the aim to strengthen local hospital collaborations and concentrate complex procedures in a limited number of reference centers [1].

In 2016, the French Public Health law no 2016-41 for the improvement of the healthcare system made it compulsory for hospitals to join a Regional Hospital Group (RHG). This law was followed by the creation of 136 collaborative hospital networks, each one having a designated leader. Before this law, the regional health agencies lacked access to information regarding transfers and patient flow between hospitals. Policymakers need to be able to determine if new laws and regulations have the desired effect, and they will attempt to make amendments to the text if they have not achieved what was aimed for. However, this process presupposes some degree of knowledge about the system for which the regulation is intended. Any attempt to reform public hospital systems needs to be based on an analysis of hospital data, be it local or foreign, as the experience of other countries may provide useful models for future regulation. The analysis of hospital data may often start as a description of the hospitals that are part of the system. Hospitals can be considered as individual entities or as parts of broader networks. Previous work has shown that the number of patients shared between physicians can predict relationships between medical providers [13]. Likewise, Social Network Analysis conducted on administrative hospital data can allow the identification of relations between hospitals [14]. These relationships can be inferred from the observation of frequent patient transfers, which denote a certain degree of trust between hospitals and are a valid proxy for the ability to collaborate for the benefit of the patient [15, 16]. Direct transfers are frequent: 1 million in France in 2014 [17]. They represent 6.4% of patient admitted to Intensive Care Units in the United States [18]. They tend to have longer lengths of stay and cost more than twice as much as those not transferred, which makes them of particular interest to hospital managers [19]. Understanding the patterns of patient mobility between hospitals is a first step towards the optimization of interactions between hospitals. Although network-level variables have not been consistently associated with quality of care, an increasing number of publications suggest such a relationship [14, 16]. Integration (distributed goals) and differentiation (coordination of specialization) are frequent in efficient organizations [20]. However, these properties have seldom been studied in real-world healthcare systems [21, 22], despite recent work calling for such studies [23]. The characteristics of hospital transfers need to be identified to ascertain how these transfers occur in relation to integrated hospital networks. The objective of this study was to use unsupervised learning methods and social network analysis to identify clusters of French public hospitals according to their characteristics and their role in the national hospital network, by taking into account patient transfers within Regional Hospital Groups in the clustering algorithm. A secondary objective was to describe patterns of patient mobility between these hospitals.

## Methods

### Data source

We used data from the French administrative hospital database. French public and private hospitals are financed through a Diagnosis-Related Group (DRG)-based prospective payment system [24]. The source database includes all hospital discharges (public and private) on the entire territory of France including overseas. The database contains information on the patients’ diagnoses during their hospital stay, encoded using the International Classification of Diseases, 10th Edition (ICD-10). Hospital stays are classified in diagnosis groups (Catégories Majeures Diagnostiques, CMD) and then in DRGs according to ICD-10 principal and secondary diagnosis codes, surgical and non-surgical procedures (Classification Commune des Actes Medicaux, CCAM) [25] and the patients’ age. As the database is used for billing hospital stays, it is subjected to numerous local and national quality controls including regular inspections by officially appointed medical information specialists. This ensures that the data stay comprehensive and accurate.

In this database, hospital stays are classified using DRG codes. However, there are more than 600 DRGs with differing severity subclasses, which makes them unpractical for the realization of summary statistics. To solve this problem, we used a higher-level classification. The Activity Domains classification assigns Activity Domains to subsets of DRGs. It is curated by the French national agency in charge of medical information [26].

Hospitals with less than 500 stays per year in Medicine, Surgery and Obstetrics were excluded. Some Regional Hospital Groups with particular geographic situations (e.g. overseas) or specializing in psychiatry (without patients in Medicine, Surgery and Obstetrics) were also excluded (list in Appendix 1). The hospitals situated inside Paris are grouped under a single legal entity responsible for over 916,000 stays (“Assistance Publique - Hôpitaux de Paris”). As they were exempt from participating in a Regional Hospital Group during the study period, they were also excluded. Patient transfers with a length of stay < 48 h in the receiving hospital were excluded. Invalid stays (due to errors in Diagnosis Related Groups or invalid patient anonymization number) and stays for iterative treatments were excluded.

### Ethical and regulatory considerations

The study was declared to the French registry of studies using healthcare data (N° F20210106180024). As the study was retrospective, based on anonymized data and purely observational, it was exempt from Institutional Review Board approval according to the French Public Health Code (L1121-1, law number 2012-300, 5 march 2012).

### Statistical methods

French public hospitals were described using two complementary methods. First, a cluster analysis was conducted to identify distinctive hospital profiles; then a Social Network Analysis was conducted to understand how these profiles interacted in the hospital network.

Clusters of hospitals were determined using the k-means method. This algorithm has the advantage of being applicable to numerical data, performs well when data are of good quality, and is easy to understand [27]. The principle of this algorithm is to choose a number K of centroids that corresponds to the number of clusters. The initial centroids are chosen randomly. Each point is then assigned to the cluster of the nearest centroid. After this step, new centroids are recalculated as the center of gravity of all points that belong to each cluster. This process is repeated until convergence is achieved. The clustering algorithm was applied to public hospitals with an activity in Medicine, Surgery and Obstetrics (MSO) in metropolitan France (excluding oversea territories) for year 2016.

The active variables included in the algorithm were:

- The number of stays in each hospital during the study period;
- The effective number [28] of activity domains;
- A mobility index.

The variables to be included in the algorithm were chosen following a preliminary exploratory analysis of the relationships between hospital-level variables. The number of stays in each hospital was included in the clustering algorithm because it was highly associated with numerous other hospital-level characteristics such as the number of beds and the number of medical and non-medical staff members. This variable can be used as a proxy for the size of the hospital, while also conveying additional information on its activity. The number of activity domains further describes the activity performed in each hospital, by characterizing the diversity of hospital stays. Finally, the mobility index was used as a network-level variable that summarizes the position of the hospital in relation to other hospitals of the same group. Together, these variables were chosen because they captured different aspects of the functioning of a hospital at the hospital level and at the network level, and because they could be expressed as numerical variables, which is a prerequisite for inclusion in the clustering algorithm. The number of variables was limited to three to improve clarity and interpretability, and because this allows a data visualization in two dimensions which makes it possible to quickly understand the key characteristics of the system.

The effective number of activity domains was calculated by using a diversity index derived from Simpson [28, 29] applied to the Activity Domains of stays produced by the hospital during the year. This method was chosen because using the absolute number of Activity Domains would result in an overestimation of the diversity of activities performed by hospitals on a regular basis. For instance, it would be misleading to say that a hypothetical hospital having 500 stays for activity “A”, 3 stays for activity “B”, and 2 stays for activity “C” performs three distinct activities on a regular basis.

The effective diversity derived from Simpson’s index is defined as:

1 $$ \mathbf{D}=\frac{\mathbf{1}}{\sum_{\mathbf{i}=\mathbf{1}}^{\mathbf{S}}{{\mathbf{p}}_{\mathbf{i}}}^{\mathbf{2}}} $$

With p_i_ corresponding to the proportion p of hospital stays of the type *i*. In this study, the types of hospital stays corresponded to the activity domains of stays produced at the hospital during the study period, encoded using the Activity Domain classification of the French national agency in charge of medical information. Simpson’s diversity index belongs to a subclass of indices that aim to allow the quantification of diversity. Simpson’s diversity index can be interpreted as the probability that two stays taken at random in the hospital’s activity during the year are of the same type. It has the property of favoring frequent activity domains, which means that adding a single stay with a new activity domain to a hospital will not have much impact on the value of the index.

The “effective number” of different hospital stays is derived from Simpson’s diversity index by taking its inverse [28]. It has desirable mathematical properties to quantify diversity: in a hospital practicing all types of stays in equal proportions, if the absolute number of different hospital stays doubles, the effective number of hospital stays will also double.

The mobility index used in this study was the percentage of stays that were followed by a transfer or readmission in another hospital of the same Regional Hospital Group within 90 days. These stays were called « Regional Hospital Group classifying stays ». Mobility includes transfers, which can be identified from electronic health records by comparing the entry and discharge dates of hospital stays: a transfer occurs when a patient enters a new hospital on the same day that he/she was discharged from the previous one. It is theoretically possible for a patient to be hospitalized in two different hospitals without any collaboration process taking place. As these cases occur randomly, they are not expected to be a major source of bias. Extending the delay between stays to 90 days allows us to account for the cases where hospitalization in the second hospital are programmed at a later date.

All active variables were standardized by subtracting the mean and dividing by the standard deviation to be on a comparable scale before entering the classification algorithm. The variables introduced in the clustering algorithm were not weighted.

### Finding the optimal number of clusters

There are several methods to determine the optimal number of clusters, each with advantages and drawbacks. In this study, the number of clusters was determined by using a selection of the various methods available and applying a majority rule. Thirty methods included in the *NbClust* R package were used, and the number of clusters most frequently designed as optimal among these methods was chosen as our number of clusters. The number of clusters was then verified by using the elbow method.

### Description of hospital clusters and their activity domains

The types of stays that constituted the activity of the hospital clusters identified by the classification algorithm were described using the Activity Domains classification [26]. The number of stays was calculated for each activity domain in each cluster. To show the differences between the distributions of activity in each cluster, the percentage of activity for each domain was calculated. The denominator for the calculations of these percentages was the sum of all stays in each cluster.

### Social network analysis of patient mobility

A Social Network Analysis (SNA) was performed to visualize the relationships between hospitals identified by studying patient transfers. Patient mobility was represented by the edges of a directed graph. The hospitals constituted the vertices of the graph. To facilitate the interpretation and readability of the graph, relations with less than 100 patients for year 2016 were excluded as not significant. Social network metrics were calculated using the *igraph* package available on the Comprehensive R Archive Network (CRAN). The assortativity degree measures the tendency of graph elements to associate with elements with a similar degree [30]. Transitivity is the tendency of elements that are connected together to share mutual connections. Edge density is the ratio of the number of existing edges by all possible edges. Data analysis was performed using R software version 4.0.2 (www.R-project.org).

## Results

**Fig. 1:**
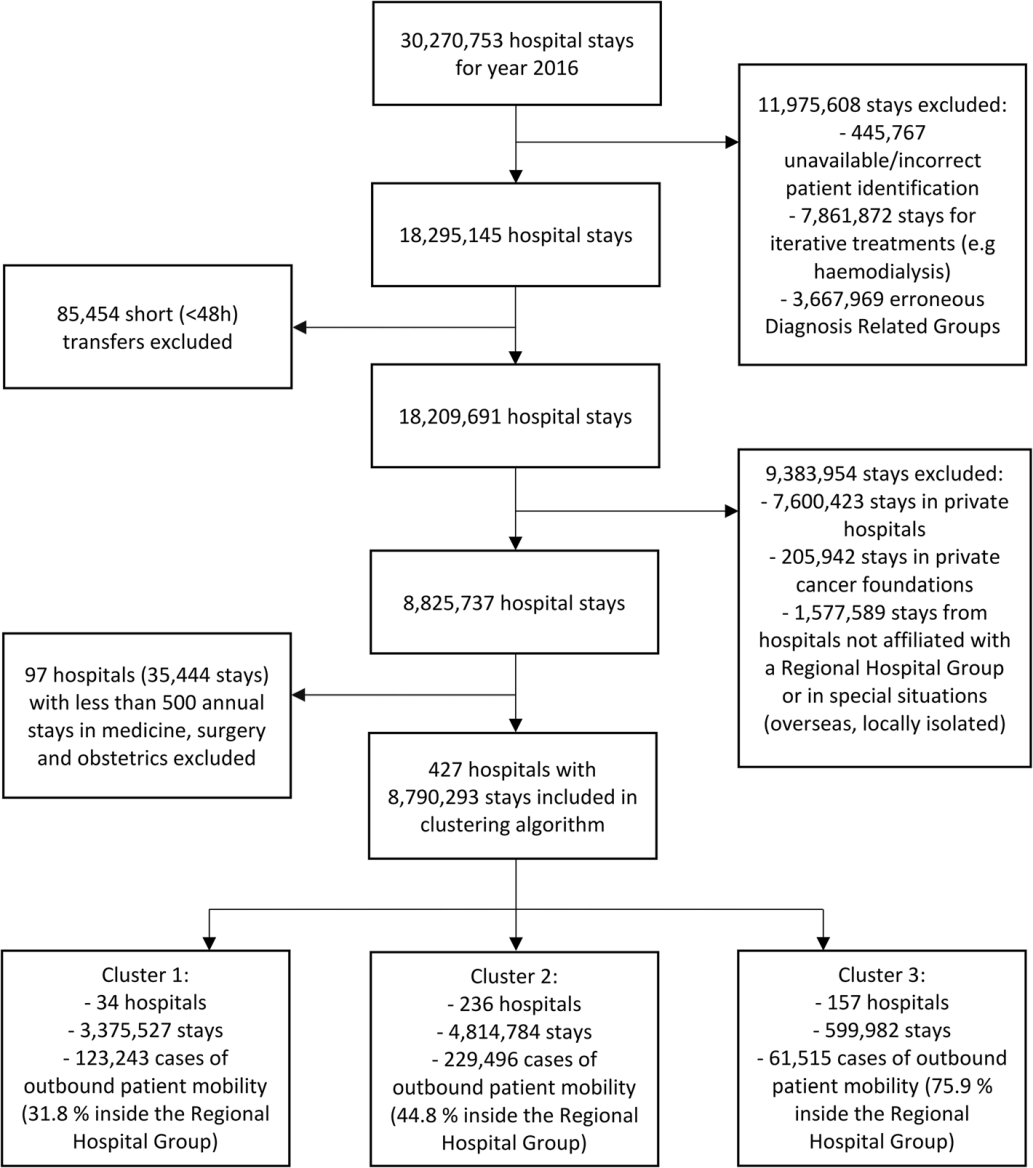
Flow chart of the study

A total of 427 hospitals representing 8,790,293 stays were included in the clustering algorithm. The flow chart of the study is presented in Fig. 1. Due to their specific situations, 39 public hospitals were excluded (6.2% of all public hospitals registered in 2016). There were 414,254 cases of patient mobility in the hospitals considered for this study. The proportion of mobility that occurred within 0, 1, 7, 30, and 60 days of the index stay was 43.6, 46.0, 54.1, 73.0 and 88.7% respectively. Overall, only 45.5% of the mobility occurred within Regional Hospital Groups (53.6% if only hospital relationships with ≥100 patients were considered).

### Number of hospital activity domains

**Fig. 2:**
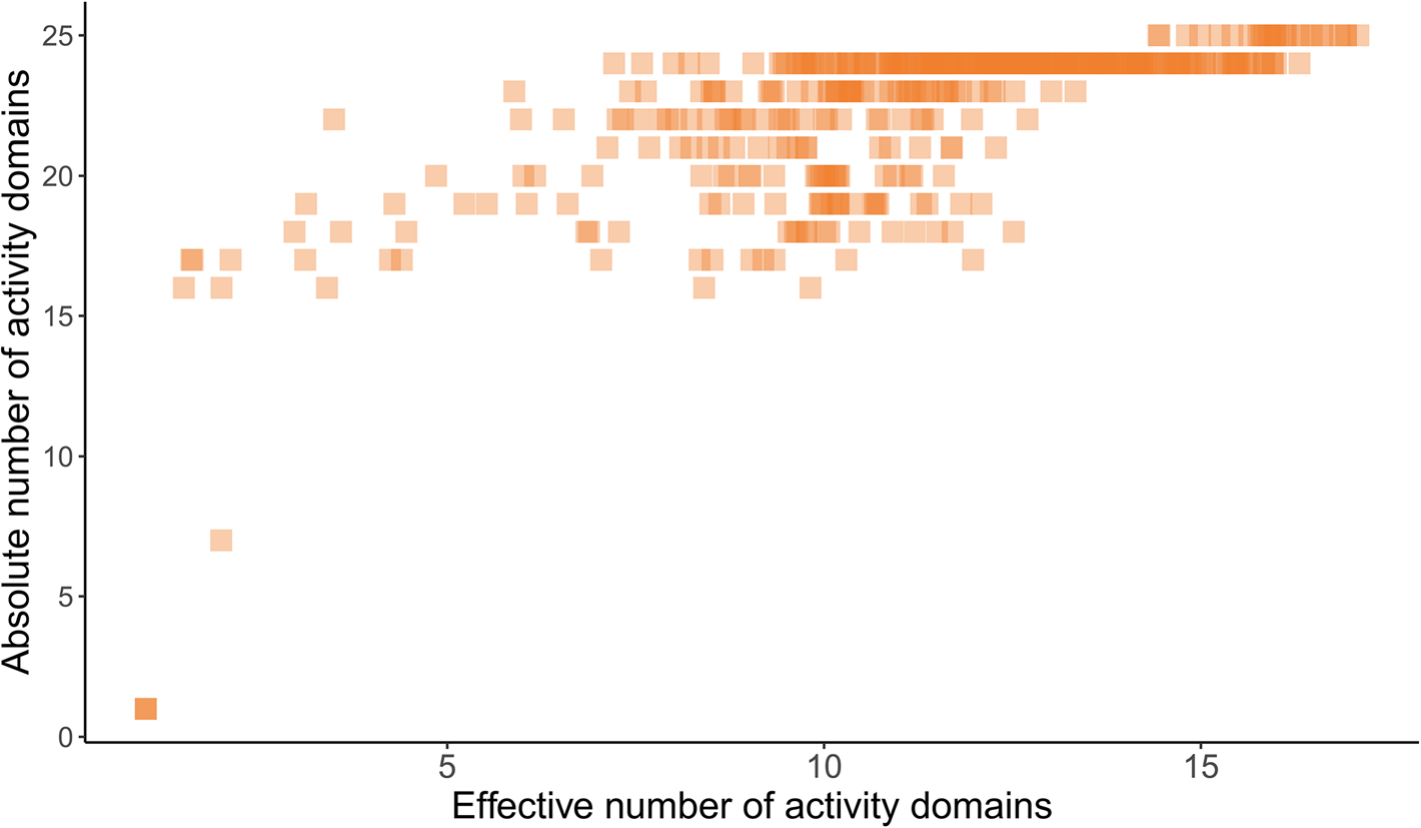
Absolute and effective numbers of activity domains per hospital

The relationship between absolute and relative number of activity domains is shown in Fig. 2. As predicted, the effective number of activity domains appeared more adequate than the absolute number of activity domains for differentiating the diversity of hospital activity. There was a ceiling effect for the absolute number of activity domains, with several hospitals attaining the highest values. Ceiling effects occur when a high proportion of participants in an experiment attain the maximum score, making them seem similar despite the existence of differences inside the group. This supports our choice of preferring to use the effective rather than absolute number of activity domains, because being spared by the ceiling effect the effective number of activity domains is able to further distinguish how varied these hospitals are in terms of diversity of the performed activities. An exploratory analysis showed that omitting the effective number of activity domains variable in the clustering algorithm changed the assigned cluster of 16.0% of the hospitals.

### Number of clusters

**Fig. 3:**
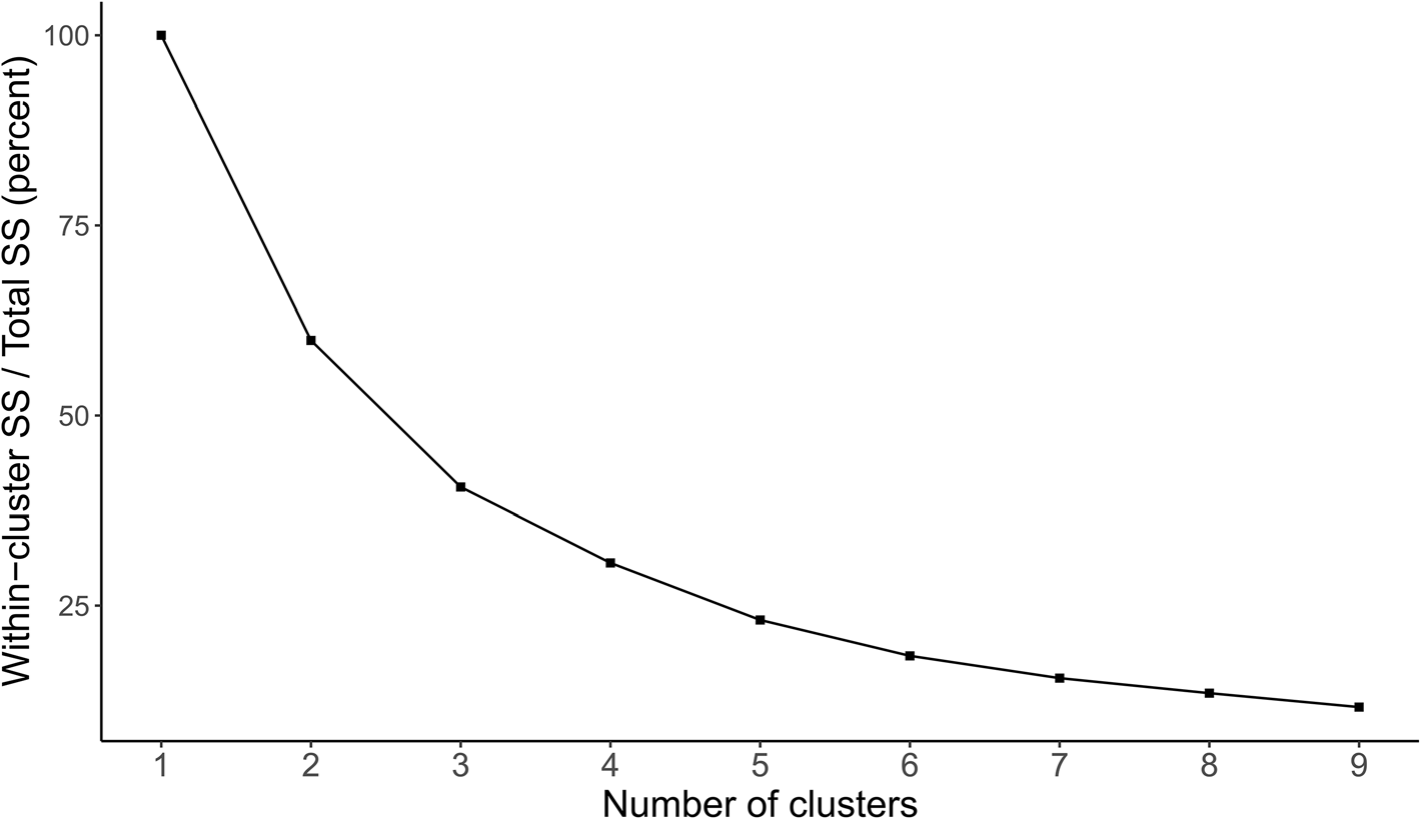
Cluster separation according to the numbers of groups in the clustering algorithm. SS: Sum of Squares

Seven out of 30 clustering separation metrics recommended three as the number of clusters that conveyed most information. This is consistent with the aspect of the elbow plot, which shows that the additional contribution to the percentage of variance explained was smaller after three clusters (Fig. 3). Following the elbow plot and the majority rule, the number of clusters was set to three.

### Hospital profiles

The hospital profiles identified by the k-means algorithm are presented in Table 1.

**Table 1:** Hospital profiles identified by the k-means algorithm

Group	Group 1 (***n*** = 34)	Group 2 (***n*** = 236)	Group 3 (***n*** = 157)
**Number of stays per hospital – median [Q1 - Q3]**^**a**^	82,108 [69,004 – 117,774]	18,913 [12,410 – 26,689]	2337 [1195 – 5285]
**Number of beds – median [Q1 - Q3]**	1129 [968 – 1431]	258 [164 - 377]	48 [30 - 80]
**Regional Hospital Group classifying stays (%)**^**b**^ **Mean (standard deviation - SD)**	2 (1)	4 (2)	11 (5)
**Regional Hospital Group leader – n (%)**	34 (100.0)	88 (37.3)	0 (0.0)
**Absolute number of activity domains** **Mean (standard deviation - SD)**	24.74 (0.45)	23.61 (1.01)	19.94 (3.65)
**Effective number of activity domains – Mean (standard deviation SD)**	15.86 (0.70)	12.26 (1.72)	8.63 (2.73)
**Degree centrality (undirected) – median [Q1 – Q3]**	252 [217 - 305]	102 [68 - 134]	29 [18 - 45]
**Strong edge (> 100 patients) degree centrality – median [Q1 – Q3]**	19 [13 - 24]	4 [3 - 6]	2 [2 - 2]
**Inbound strength (incoming mobility) – median [Q1 – Q3]**	4591 [2991 – 5631]	741 [431 – 1075]	292 [203 - 410]
**Outbound strength (exterior mobility) – median [Q1 – Q3]**	3307 [2432 – 4111]	946 [620 – 1198]	349 [152 - 551]

^a^Hospital stays in Medicine, Surgery and Obstetrics (MSO), for year 2016 in metropolitan France (excluding oversea territories), with quality control code equal to 0 (valid stays), excluding stays with an undefined Diagnosis Related Group (Major Diagnostic Category 90) and excluding patient transfers with a length of stay < 48 h in the receiving hospital

^b^Stays that were followed by subsequent stays in another hospital of the same Regional Hospital Group within 90 days

**Fig. 4:**
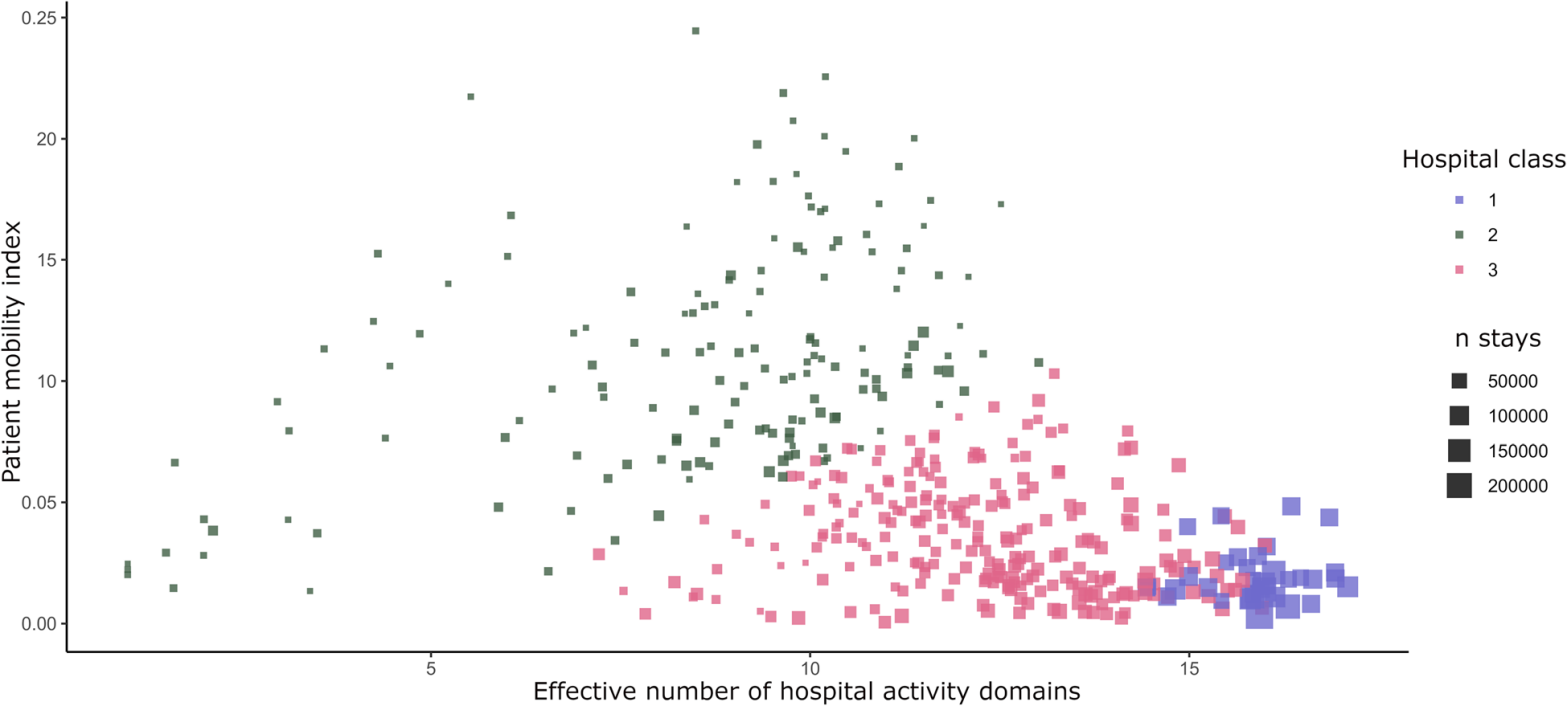
Clustering algorithm results displayed in the active variable space. The patient mobility index indicates the mobility of patients within the same Regional Hospital Group. A high mobility index means that patients often move to other hospitals of the same Regional Hospital Group

Patients’ mobility towards other hospitals of the Regional Hospital Group was inversely proportional to the total number of stays per hospital (Fig. 4). The hospitals included in each cluster had different activity profiles (Table 2). Cardiovascular catheterization, the treatment of cancer, neurological disease and organ transplantation were preferentially performed in Group 1 hospitals. Conversely, digestive and pneumology activity domains represented a higher proportion of stays in Group 3 and 2 hospitals.

**Table 2:** Activity domains of the hospital profiles

Activity domain	Group 1 – N (% of stays)	Group 2 – N (% of stays)	Group 3 – N (% of stays)
**Digestive system**	371,553 (11.0)	728,168 (15.1)	105,647 (17.6)
**Orthopaedics, trauma care**	218,590 (6.5)	354,687 (7.4)	43,024 (7.2)
**Complex trauma**	5663 (0.2)	2835 (0.1)	249 (0)
**Diseases of the bone and joint**	103,486 (3.1)	109,229 (2.3)	17,433 (2.9)
**Diseases of the nervous system**	331,825 (9.8)	317,859 (6.6)	38,435 (6.4)
**Diagnostic and therapeutic vascular catheterisms**	127,284 (3.8)	104,315 (2.2)	544 (0.1)
**Cardiolovascular medicine**	244,072 (7.2)	392,896 (8.2)	65,098 (10.9)
**Pulmonology**	218,533 (6.5)	363,264 (7.5)	52,243 (8.7)
**Ear, nose and throat. Oral medicine**	124,211 (3.7)	171,502 (3.6)	14,636 (2.4)
**Ophtalmology**	107,057 (3.2)	114,451 (2.4)	11,575 (1.9)
**Gynecology – breast diseases**	80,690 (2.4)	101,535 (2.1)	7172 (1.2)
**Obstetrics**	217,830 (6.5)	467,071 (9.7)	39,593 (6.6)
**Newborns, perinatal period**	132,451 (3.9)	302,957 (6.3)	23,298 (3.9)
**Urology, nephrology, male genital system**	200,518 (5.9)	270,610 (5.6)	30,623 (5.1)
**Hematology**	95,755 (2.8)	97,065 (2)	13,219 (2.2)
**Chemotherapy, radiotherapy, excluding iterative treatments**	70,488 (2.1)	40,885 (0.8)	1797 (0.3)
**Infectious disease (including HIV)**	35,028 (1)	47,911 (1)	4548 (0.8)
**Endocrinology**	125,642 (3.7)	139,507 (2.9)	14,769 (2.5)
**Skin and subcutaneous tissue**	87,844 (2.6)	105,833 (2.2)	14,389 (2.4)
**Burns**	6161 (0.2)	2197 (0)	214 (0)
**Psychiatry**	88,586 (2.6)	110,198 (2.3)	21,941 (3.7)
**Toxicology, alcohol-related disease**	82,024 (2.4)	153,170 (3.2)	33,064 (5.5)
**Chronic pain, palliative care**	37,994 (1.1)	48,723 (1)	8779 (1.5)
**Organ transplantation**	3844 (0.1)	1 (0)	0 (0)
**Transversal disease management and follow-up**	257,172 (7.6)	266,162 (5.5)	37,567 (6.3)

### Social network analysis

The Social Network Analysis was conducted in two stages: Stage 1 kept only the strongest links between hospitals (at least 100 patients in either direction). Stage 2 considered all links indiscriminately.

**Fig. 5:**
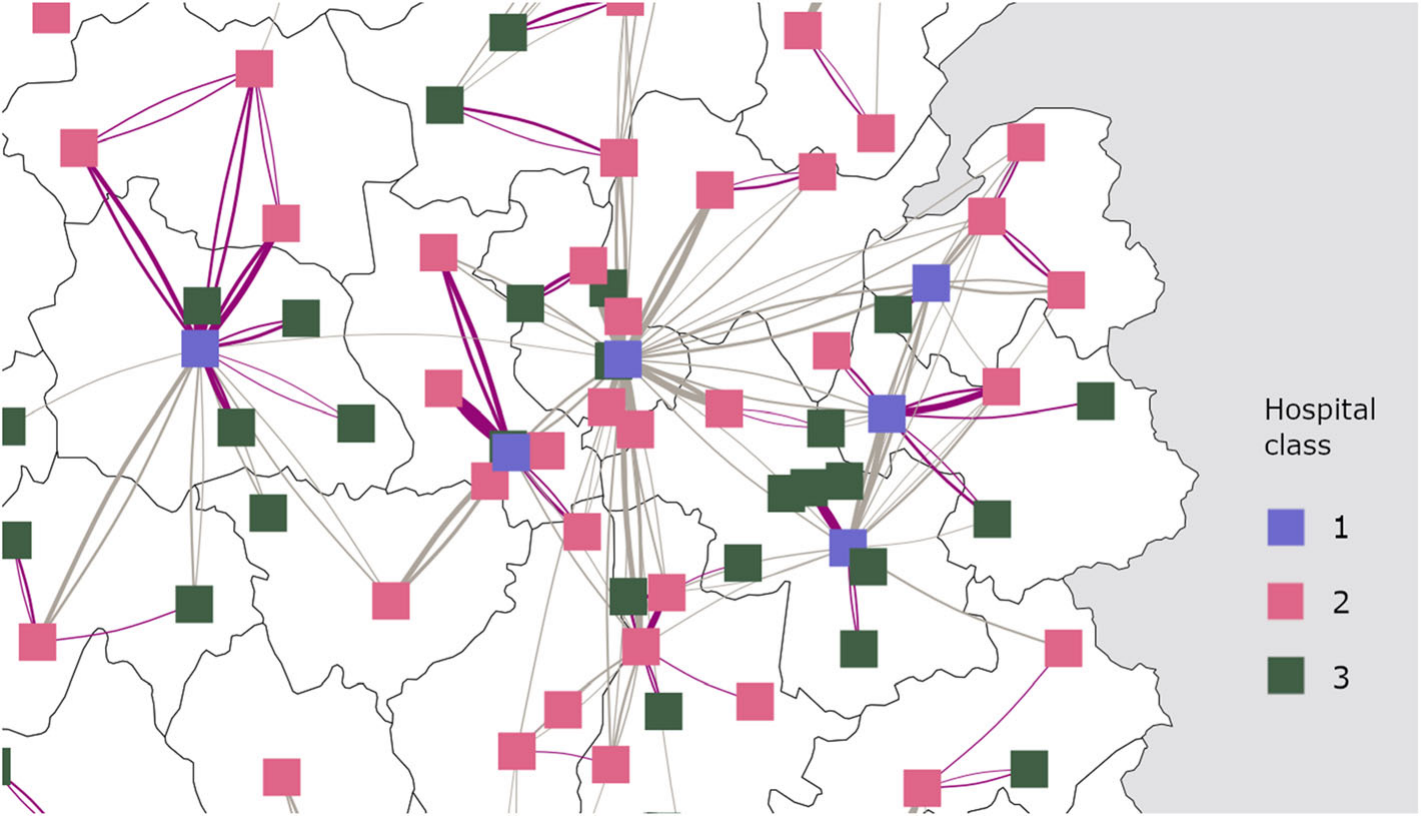
Social Network Analysis (SNA) of public hospitals in France centered on the Auvergne-Rhône-Alpes region. The hospitals are mapped to vertices colored by their class. The edges represent patient flow. Edges colored in purple are between hospitals of the same Regional Hospital Group. Edges are limited to relations with > 100 patients

When restricted to strong edges (> 100 patients per directed edge), the network had an assortativity degree of − 0.263. The unrestricted network had a more neutral assortativity degree of − 0.043. Overall, this is consistent with the fact that the mobility of patients was preferentially directed towards hospitals with different profiles. The larger hospitals acted as reference care providers and performed treatments for patients that would have been difficult to manage in small hospitals (Fig. 5). The transitivity of the strong edges (> 100 patients) network was 18.0% (39.0% for the overall network considering all edges), which is also consistent with a network made of multiple loosely connected centralized islets. The overall edge density on the study territory was 10.6% (0.6% when restricting to edges > 100 patients).

Mobility patterns in hospital clusters are presented in Table 3. Group 1 mainly acted as a recipient of patients and had the highest incoming patients per hospital ratio (Table 3). However, due to the large number of hospitals included in Group 2, the raw number of patients received in Group 2 (193,938) was higher than in Group 1 (166,953). Group 3 hospitals referred to Group 2 hospitals before referring to Group 1 hospitals, perhaps because Group 1 hospitals were less frequent.

**Table 3:** Mobility types between clusters for year 2016

Initial cluster (sending hospital)	Next cluster (receiving hospital)	Number of patients	Percentage from departure group	Percentage of overall total
G1				
	G2	79,928	65%	19%
	G1	22,665	18%	5%
	G3	20,650	17%	5%
G2				
	G1	118,601	52%	29%
	G2	84,322	37%	20%
	G3	26,573	12%	6%
G3				
	G2	29,688	48%	7%
	G1	25,687	42%	6%
	G3	6140	10%	1%

## Discussion

Using unsupervised learning methods, we identified three hospital classes with different degrees of network centrality in French public hospital data for year 2016.

The hospitals assigned to Group 1 deliver most of the country’s experimental (quaternary) and specialized (tertiary) care. They do not need to transfer patients to other hospitals of the same Regional Hospital Group because they themselves host a sufficient array of healthcare services. Patient-sharing is easier within these teaching hospitals, corridor distance being only one of the factors involved [31]. These hospitals have the potential to act as information brokers and set quality of care standards [32]. However, their size can make it difficult for them to be located in the most central urban areas of their cities [33]. On the other hand, Group 2 and 3 hospitals often need to transfer patients with complex needs to these centers. The situation is similar in the United States, where between one third and one half of myocardial infarction patients will be transferred from a non-revascularization center to a center equipped for the procedure [34]. In the future, smaller hospitals are expected to develop the activities for which they used to transfer their patients, and larger hospitals are expected to develop innovative activities not available elsewhere [35].

Despite studies suggesting that hospital networks occur endogenously out of previous relationships [15], our data shows that hospital collaboration networks are often different from what would be expected based on administrative boundaries such as region limits (only 45.5% of the mobility occurring within a Regional Hospital Group). In his 1994 study, Bolland [36] had observed that referrals and planning represent two dimensions that are largely non-overlapping in a variety of American counties. Instead, the observed mobility relationships between hospitals often involve the referral of patients to reference hospitals regardless of their belonging (belonging to the same regional group or not). This is a problem, because it suggests that the hospitals that exchange patients and would ideally work together as a unified infrastructure (“basic systems and services that are reliable, standardized, and widely accessible”) could lack the common management and unity of purpose (no formal embedding) [37] to conduct patient transfers efficiently [34]. It is well known that the negotiation process for a transfer can be difficult [38], and this difficulty is expected to be greater if there are administrative barriers between hospitals [5]. Likewise, these transfers “across borders” could result in difficulties of communication [39, 40].

On the other hand, networks that are open to outside influence are closer to the unconstrained network structure that shows the most favorable profile for the diffusion of innovation [41]. Our observations are consistent with data obtained by Le Meur [42] and Rankin [43], where hospital networks have a high degree of centralization. At a lower level, this could be linked with data indicating that well-connected doctors have a positive impact on patient experience [44–47]. This centralized type of organization is consistent with good quality of care. Less centralized networks are likely the result of capacity overload rather than the need to transfer to more adequate facilities [48]. In our study, the most central hospitals had a higher proportion of cancer patients, a finding consistent with data from Orange County (California, USA) [49]. They are more likely to have neurology wards and trauma centers, to perform organ transplantation, and to be affiliated to medical schools, as was also noted by a nationwide American study focusing on critical care patients [50]. These hospitals are expected to have lower readmission rates (as shown in an Italian study) [16], and lower mortality and length of stay especially for the more severe cases [51]. Therefore, referral to these hospitals can be life-saving [52] if improving the quality of care in smaller hospitals is not possible. This shows that the structure of hospital networks could be similar across very different countries. The tendency to send patients to these hospitals could also be exacerbated by rivalry between smaller hospitals, due to niche overlap [53]. Lomi et al. have contended that hospitals that meet in a restricted spatial setting (which ordinarily generates rivalry) face new opportunities for collaboration due to the institutional nature of hospitals and to mutual forbearance, a tendency to spontaneously specialize [54]. However in the case of the French network this rarely occurs, possibly due to the rural setting of directly competing hospitals and the similarity of their case mixes [15, 55]. Thus, contrary to technology firms, hospitals that share the same case mix are less likely to form bonds [56]. This could explain the findings of a study suggesting that competition does not always favor productivity in hospitals [57]. The temptation to treat all patients in Group 1 hospitals would conduct to a situation experienced in the United States during the 1980s: a decrease of commitment of smaller hospitals, with an excessive tendency to transfer patients, which led to saturation of the larger centers [58]. Nonetheless, based on our results, policymakers could suggest a revision of the contracts binding public hospitals groups, with an emphasis on linking smaller hospitals to larger referral centers, in order to make the composition of these groups consistent with observed collaboration patterns, which could improve the overall coordination of care. Drawing on the aforementioned past experience in the USA, this should be done in a manner that preserves capability for urgent care in smaller centers.

Social Network Analysis has rarely been applied in the hospital setting at the national level, yet this method could be useful in guiding data-based interventions [21, 59]. For instance, it could help in predicting the spread of hospital-acquired infections [60–62]. Our study has some limitations: although we included the vast majority of French public hospitals in this nationwide study, some hospitals were excluded due to their specific geographic situation (e.g. Hospitals of Paris, overseas hospitals). Although these centers represented less than 10% of public hospitals, additional studies are needed to evaluate their patterns of patient mobility before any change of policy is implemented.

In further studies, characteristics associated with network strength could be studied by using gravity models, which account for the distance between hospitals in addition to their size [63, 64]. Future research also needs to ascertain if the national hospital network displays integration and differentiation [23] through the elaboration of formal healthcare pathways, or if patient mobility is due to other reasons like differences in quality of care. Further studies could explore the specific diseases and types of activities involved in patient mobility or transfers.

## Supplementary Information

**Additional file 1.**

## Data Availability

The data that support the findings of this study are available from the French national agency in charge of medical information, but restrictions apply to the availability of these data, which were used under the MR005 legal framework, and so are not publicly available. Aggregated and anonymized data are however available from the authors upon reasonable request with permission by the French national agency for medical information.
