# Supplementary material for: Making sense of the French public hospital system: a network-based approach to hospital clustering using unsupervised learning methods
Source: BMC Health Serv Res. 2021 Nov 17;21:1244. doi: 10.1186/s12913-021-07215-4 (PMC8600901; doi:10.1186/s12913-021-07215-4)
Supplement: Supplementary file 1 — Additional file 1. [file 12913_2021_7215_MOESM1_ESM.pdf]

Appendix 1

Excluded Regional Hospital Groups

Name	Reason for exclusion
Nord Franche-Comté	Locally isolated regional hospital group
Psychiatrie Doubs-Jura	Psychiatry-oriented regional hospital group (administrative field outside of Medicine, Surgery and Obstetrics)
Haute-Saône	Locally isolated regional hospital group
Sud Côte d'Or	Locally isolated regional hospital group
Île du nord	Overseas
GHT de la Guadeloupe	Overseas
GHT de la Guyane	Overseas
Psychiatrie 59-62	Psychiatry-oriented regional hospital group (administrative field outside of Medicine, Surgery and Obstetrics)
77 Nord	Locally isolated regional hospital group
94 Nord	Locally isolated regional hospital group
Psy Sud Paris	Psychiatry-oriented regional hospital group (administrative field outside of Medicine, Surgery and Obstetrics)
Paris psychiatrie et neurosciences	Psychiatry-oriented regional hospital group (administrative field outside of Medicine, Surgery and Obstetrics)
Provins Est Seine-et-Marne	Locally isolated regional hospital group
Cotentin	Locally isolated regional hospital group
Océan Indien	Overseas
